# The Use of SNP Markers for Linkage Mapping in Diploid and Tetraploid Peanuts

**DOI:** 10.1534/g3.113.007617

**Published:** 2013-11-08

**Authors:** David J. Bertioli, Peggy Ozias-Akins, Ye Chu, Karinne M. Dantas, Silvio P. Santos, Ediene Gouvea, Patricia M. Guimarães, Soraya C. M. Leal-Bertioli, Steven J. Knapp, Marcio C. Moretzsohn

**Affiliations:** * Departamento de Genética/IB, Universidade de Brasília, Brasília, DF, 70910-900, Brazil; † Department of Horticulture, University of Georgia, Tifton, Georgia 31973; ‡ Embrapa Recursos Genéticos e Biotecnologia, Brasília, DF, 70770-900, Brazil; § Universidade Católica de Brasília, Brasília, DF, 70790-160, Brazil; ** Institute of Plant Breeding, Genetics, and Genomics, University of Georgia, Athens, Georgia 30602

**Keywords:** *Arachis*, breeding, genotyping, markers, wild

## Abstract

Single nucleotide polymorphic markers (SNPs) are attractive for use in genetic mapping and marker-assisted breeding because they can be scored in parallel assays at favorable costs. However, scoring SNP markers in polyploid plants like the peanut is problematic because of interfering signal generated from the DNA bases that are homeologous to those being assayed. The present study used a previously constructed 1536 GoldenGate SNP assay developed using SNPs identified between two *A. duranensis* accessions. In this study, the performance of this assay was tested on two RIL mapping populations, one diploid (*A. duranensis* × *A. stenosperma*) and one tetraploid [*A. hypogaea* cv. Runner IAC 886 × synthetic tetraploid (*A. ipaënsis* × *A. duranensis*)^4×^]. The scoring was performed using the software GenomeStudio version 2011.1. For the diploid, polymorphic markers provided excellent genotyping scores with default software parameters. In the tetraploid, as expected, most of the polymorphic markers provided signal intensity plots that were distorted compared to diploid patterns and that were incorrectly scored using default parameters. However, these scorings were easily corrected using the GenomeStudio software. The degree of distortion was highly variable. Of the polymorphic markers, approximately 10% showed no distortion at all behaving as expected for single-dose markers, and another 30% showed low distortion and could be considered high-quality. The genotyped markers were incorporated into diploid and tetraploid genetic maps of *Arachis* and, in the latter case, were located almost entirely on A genome linkage groups.

The peanut (*Arachis hypogaea* L.) is a grain legume and oil crop of very significant importance in tropical and subtropical regions of the world. It has a narrow genetic base because of its recent allotetraploid origin (Halward *et al.* 1991; Kochert *et al.* 1991). This limits improvement for some agronomically important traits and has hindered the development of genetic maps and the application of molecular breeding.

Cytogenetic, phylogeographical, and molecular evidence indicate that the most probable A and B genome donors to *A. hypogaea* are *A. duranensis* and *A. ipaënsis*, respectively (Kochert *et al.* 1996; Seijo *et al.* 2007; Robledo and Seijo 2010; Moretzsohn *et al.* 2013). It is estimated that the A and B genomes diverged approximately 3–3.5 million years ago (Nielen *et al.* 2012; Moretzsohn *et al.* 2013), much more recently than the subgenomes of cotton or soybean, which diverged approximately 6.7 and 13 million years ago, respectively (Senchina *et al.* 2003; Schmutz *et al.* 2010).

From genetic maps, it can be seen that the molecular marker order in the A and B genomes is conserved, with only a few major rearrangements between them (Burow *et al.* 2001; Moretzsohn *et al.* 2009; Guo *et al.* 2012; Shirasawa *et al.* 2013). This gives further support to the evidence of recent divergence of the two genome components. However, it is also evident that the A and B genomes have diverged in a very significant way: cultivated peanut behaves genetically as a diploid and almost all pairing of chromosomes during meiosis is bivalent (Smartt 1990).

The development of molecular markers for peanut has followed the technical trends of the times. The first studies were based on isozymes and proteins (Krishna and Mitra 1988; Lu and Pickersgill 1993), followed by restriction fragment length polymorphism (RFLP) (Kochert *et al.* 1991, 1996), random amplified polymorphic DNA (RAPD) (Halward *et al.* 1991; Hilu and Stalker 1995), amplified fragment length polymorphism (AFLP) (He and Prakash 1997; Tallury *et al.* 2005), and, more recently, microsatellite markers (Hopkins *et al.* 1999; Macedo *et al.* 2012) and MITE-based markers (Shirasawa *et al.* 2013). Generally, these markers have shown a trend toward becoming more informative, cost-effective, and easier to use. However, all these marker types show low polymorphism in cultivated peanut germplasm. This has stimulated the interest in wild species as sources of genetic polymorphism for mapping and as sources of new phenotypic traits such as disease resistance in breeding programs (Bertioli *et al.* 2011).

Currently, microsatellites, being co-dominant and easy to score in the tetraploid genome, are the markers of choice for molecular breeding and genetic studies. For foreground selection, in which flanking markers are used to track QTL in breeding programs, microsatellite markers are an excellent option (Cheema *et al.* 2008; Wang and Chee 2010; Foncéka *et al.* 2012). However, for background selection, in which a larger number of markers are needed to provide an overview of the different parental contributions to a progeny’s genome, the cost and time needed for microsatellite assays are high or even prohibitive.

Single nucleotide polymorphism (SNP) markers have proved very powerful in the genetic analysis of other species and useful in breeding programs. Perhaps most importantly, SNPs can be used in parallel assays, and the cost per data point is favorable compared to other marker types (when used in large-scale assays). However, SNP markers have proved very difficult to detect and apply in the cultivated peanut. This is because true SNP polymorphisms (A *vs.* A genome or B *vs.* B) are very rare and difficult to detect against the background of false A *vs.* B polymorphisms. If we consider that even very diverse peanut cultivars have diverged only a few thousand years ago, whereas the A and B genomes diverged a few million years ago, then we can expect true SNP rates to be in the region of 1000-times less frequent than false A *vs.* B rates. In addition to this difficulty of discovering SNPs are the difficulties of SNP scoring in a tetraploid genome.

Here, we report the use of SNP markers in diploid and tetraploid recombinant inbred mapping populations of *Arachis*. We anticipate that these results provide a very significant step toward the use of highly parallel SNP assays for background selections in breeding progeny derived from cultivated peanut × wild crosses.

## Materials and Methods

### Mapping populations

Recombinant inbred mapping populations were produced from unique F_1_ plants cloned by cuttings to produce enough F_2_ seeds and single-seed descent to the F_5_/F_6_ generations. The F_1_ plants were derived from the following: for the diploid population, a cross between *A. duranensis* K7988 and *A. stenosperma* V10309 (Moretzsohn *et al.* 2005; Leal-Bertioli *et al.* 2009; Bertioli *et al.* 2009); and for the tetraploid population, from a cross between *A. hypogaea* cv. Runner IAC 886 and a colchicine-induced tetraploid with amphidiploid genetic behavior, made using the most probable ancestral species of cultivated peanut (*A. ipaënsis* K30076 and *A. duranensis* V14167)^4×^ (Fávero *et al.* 2006; Fonceka *et al.* 2009; Fonceka *et al.* 2012).

### Extraction of DNA and genotyping

Total genomic DNA was extracted from young leaves using the protocol of Grattapaglia and Sederoff (1994), modified by the inclusion of an additional precipitation step with 1.2 M NaCl. DNA concentration was estimated by agarose gel electrophoresis comparing the fluorescence intensities of the ethidium bromide–stained samples to those of lambda DNA standards. Samples were further quantified with picogreen and diluted to 10–50 ng/ul.

SNP genotyping was performed using the 1536 Illumina GoldenGate array described by Nagy *et al.* (2012). This SNP array was constructed using SNP polymorphisms identified between two *A. duranensis* accessions, PI 475887 and Grif 15036. Most polymorphisms were between ESTs (1236), with the remaining being between intron sequences of predicted single copy genes (300) (Choi *et al.* 2006). As expected from the study of Nagy *et al.* (2012), not all SNP assays were functional. Of the total 1536 assays, 1054 produced marker genotypes that were incorporated into the diploid, intrapecific, *A. duranensis* A-genome map.

The calling of genotypes was performed using the software GenomeStudio version 2011.1. The software assigns GenCall scores to each data point. The GenCall score is a value between zero and one and is primarily designed to filter out failed genotypes, DNAs, and/or loci (Oliphant *et al.* 2002). Scores less than 0.2 usually indicate failed genotypes and scores more than 0.7 usually report high-quality genotypes. For this work, all GenCall scores less than 0.25 were marked as “uncalled.”

Also, the software assigns another score, the GenTrain score, to each SNP assay as a whole (compared to GenCall scores that relate to each individual data point). This score depends on the grouping of the clusters in the signal intensity plots and their relative distance from each other. This score is designed to mimic overall quality evaluations for each assay made by a human expert and, again, ranges from zero to one.

### Construction of linkage maps

Linkage maps for the two populations used in this study have already been generated (Shirasawa *et al.* 2013). The SNP markers genotyped in this study were used together with selected markers from these previous maps to further saturate the maps and to observe the behavior of SNP markers in the contrasting diploid and tetraploid genetic states. In total, 387 and 800 polymorphic markers were used for map construction in the diploid and tetraploid populations, respectively. These consisted of the following: for the diploid A genome population, 329 SNPs and 58 selected syntenic microsatellite markers; and for the tetraploid population, 394 SNPs and 406 chosen microsatellite or MITE markers. The 58 microsatellite markers were included on the diploid A genome map to enable the linkage groups to be assigned according to previously published *Arachis* maps (Moretzsohn *et al.* 2005, 2009; Leal-Bertioli *et al.* 2009; Gautami *et al.* 2012; Shirasawa *et al.* 2013). Additionally, 100 selected syntenic microsatellites were included on the A genome component of the tetraploid map to enable A–B homeolog identification. The remaining 306 microsatellites were mapped on the B genome component of the tetraploid map. Because the SNPs were developed for an A genome species (*A. duranensis*), only a few SNP markers were mapped on the B genome. Therefore, the inclusion of a higher number of microsatellite markers in the B genome component map was necessary for construction of the 10 linkage groups. Mapping populations consisted of 90 F_5_ and 89 F_6_ individuals for the diploid and tetraploid populations, respectively.

A chi-squared test was performed to test for 1:1 segregations on all scored markers. Only marker loci that did not show segregation distortion (*P* < 0.05) were used for the initial map construction to eliminate spurious linkages. The linkage analysis was performed using Mapmaker Macintosh version 2.0 (Lander *et al.* 1987). A minimum LOD score of 4.0 and maximum recombination fraction (θ) of 0.35 were set as thresholds for linkage group (LG) determination with the “group” command. The most likely marker order within each LG was estimated by the matrix correlation method using the “first order” command. Marker orders were confirmed by permuting all adjacent triple orders (“ripple” command). After establishment of the group orders, the LOD score was set to 3.0 to include additional markers in the groups. The exact position of the new markers within each group was determined by using the “try” command, which compares the maximum likelihood of each marker order after placing the markers, one by one, into every interval of the established order. In a next step, distorted markers were included using the “group” command. The new marker orders were again confirmed with the “first order” and/or “ripple” commands. Recombination fractions were converted into map distances in centimorgans using the Kosambi mapping function and the “error detection” available in Mapmaker/EXP 3.0 (Lander *et al.* 1987; Lincoln *et al.* 1992).

## Results

### Production of mapping populations

When generations were advanced, the vigor of the lines generally had a tendency to decline. In the case of the tetraploid population, this tendency was very slight and, generally, the vigor and seed set of the lines were very high. In the case of the diploid population, the loss of vigor and poor seed set were much more common. Lines of both populations contrasted in many phenotypic traits, such as flower color, height of central stem, number and length of side branches, seed size, pod morphology, resistance to foliar diseases and nematodes, and many other characteristics.

### Genotyping

From the 1536 SNP assays on the Illumina array, some detected polymorphism but others did not. Initial screening of parental DNAs indicated polymorphism for approximately 500 of the SNP assays in the diploid A genome and tetraploid mapping populations. The parentals of a B genome mapping population derived from a cross between *A. ipaënsis* and *A. magna* (Moretzsohn *et al.* 2009) were also screened, but this indicated that only approximately 100 assays may be informative.

**Figure 1:**
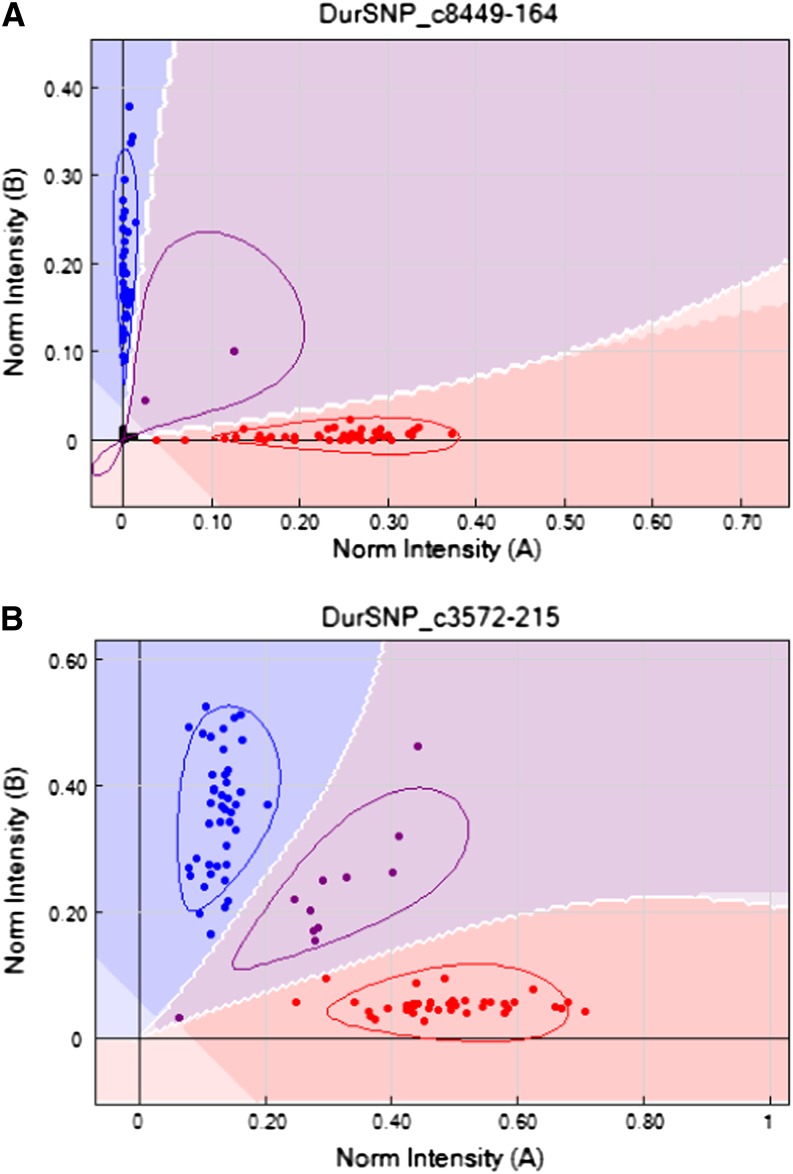
Examples of plots of signal intensities from SNP assay on the diploid population (A) and the tetraploid population after manual correction (B). Points in red and blue are homozygotes; points in purple are heterozygotes. Note how, in the tetraploid, the clustering is distorted and the separation between the genotype groups is reduced (B).

**Figure 2:**
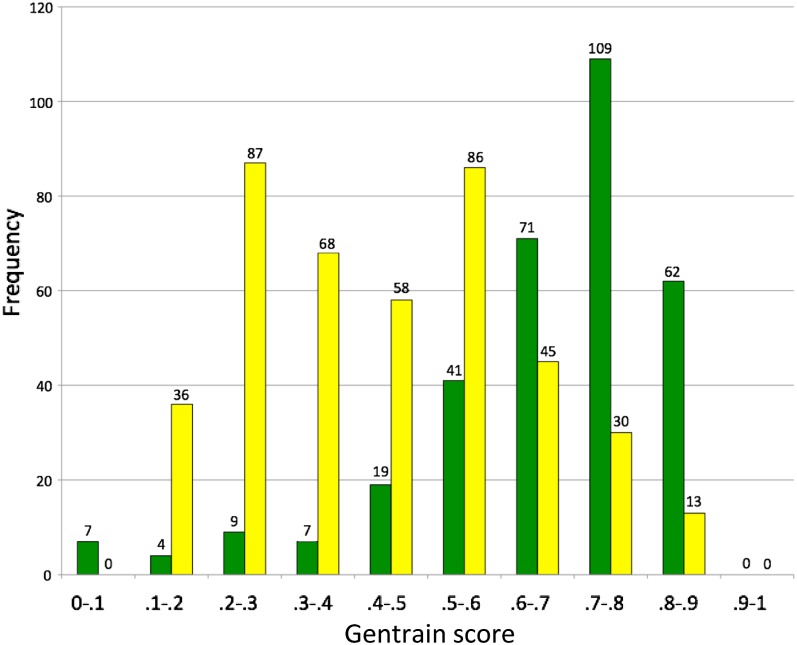
Frequency distributions in numbers of SNPs in quality score (Gentrain scores) classes for genotyping assays in the diploid (green) and tetraploid (yellow) mapping populations. The numbers of assays in each class are written above the bars. The scores for the diploid population form a single high-scoring peak, the scores for the tetraploid population form a double peak. Note that a significant number of the assays in the tetraploid population are high-quality.

In the full A genome diploid population, 329 assays were informative. Generally, separation of the genotypic states was very clear with the points lying along the “x” and “y” axes of the signal intensity plots (Figure 1A). Genotyping calling of signal intensity plots were inspected manually and only 30 needed to be manually changed. GenTrain scores ranged from 0.01 to 0.90, with a median value of 0.70 (Figure 2).

**Figure 3:**
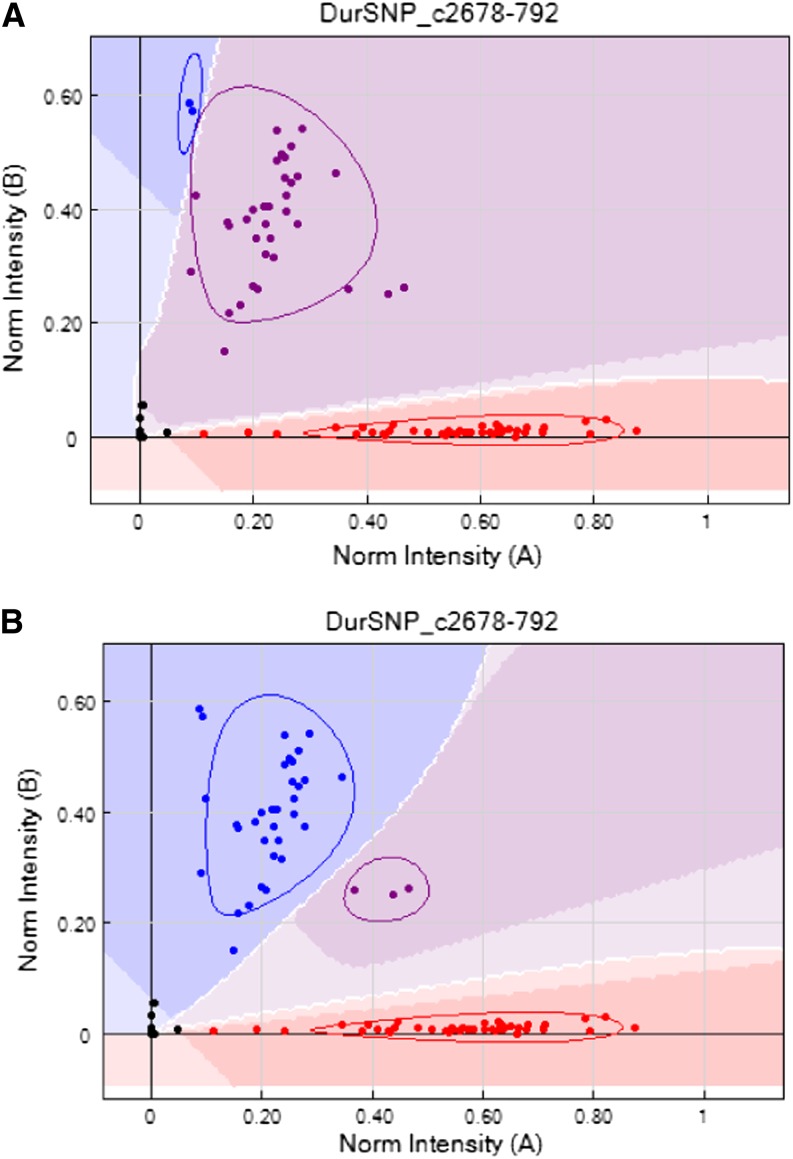
An example of a plot of signal intensities from SNP assay on the tetraploid population using default parameters (A) and after manual correction (B). Points in red and blue are called as homozygotes; points in purple are heterozygotes. Note how genotype calling using default parameters falsely assigns one of the homozygous genotypes as heterozygous.

In the tetraploid population, 394 assays were informative. Signal intensity plots were generally distorted compared to the diploid, with the points representing one allele in the homozygous state lying along one axis and points representing the other homozygous state being shifted at an angle (Figure 1B). Almost all the default parameter genotyping calls needed to be manually modified (Figure 3); only 43 were left unchanged. GenTrain scores ranged from 0.15 to 0.88, with a median value of 0.42 (Figure 2). Full genotyping information for both mapping populations is available (Supporting Information, File S1).

The frequency distributions of Gentrain scores in the diploid and tetraploid populations are distinct (Figure 2). The diploid shows a single peak at 0.7–0.8. The tetraploid shows two peaks, one at 0.2–0.3 and the other at 0.5–0.6.

### Linkage maps

#### Diploid A genome map:

**Figure 4:**
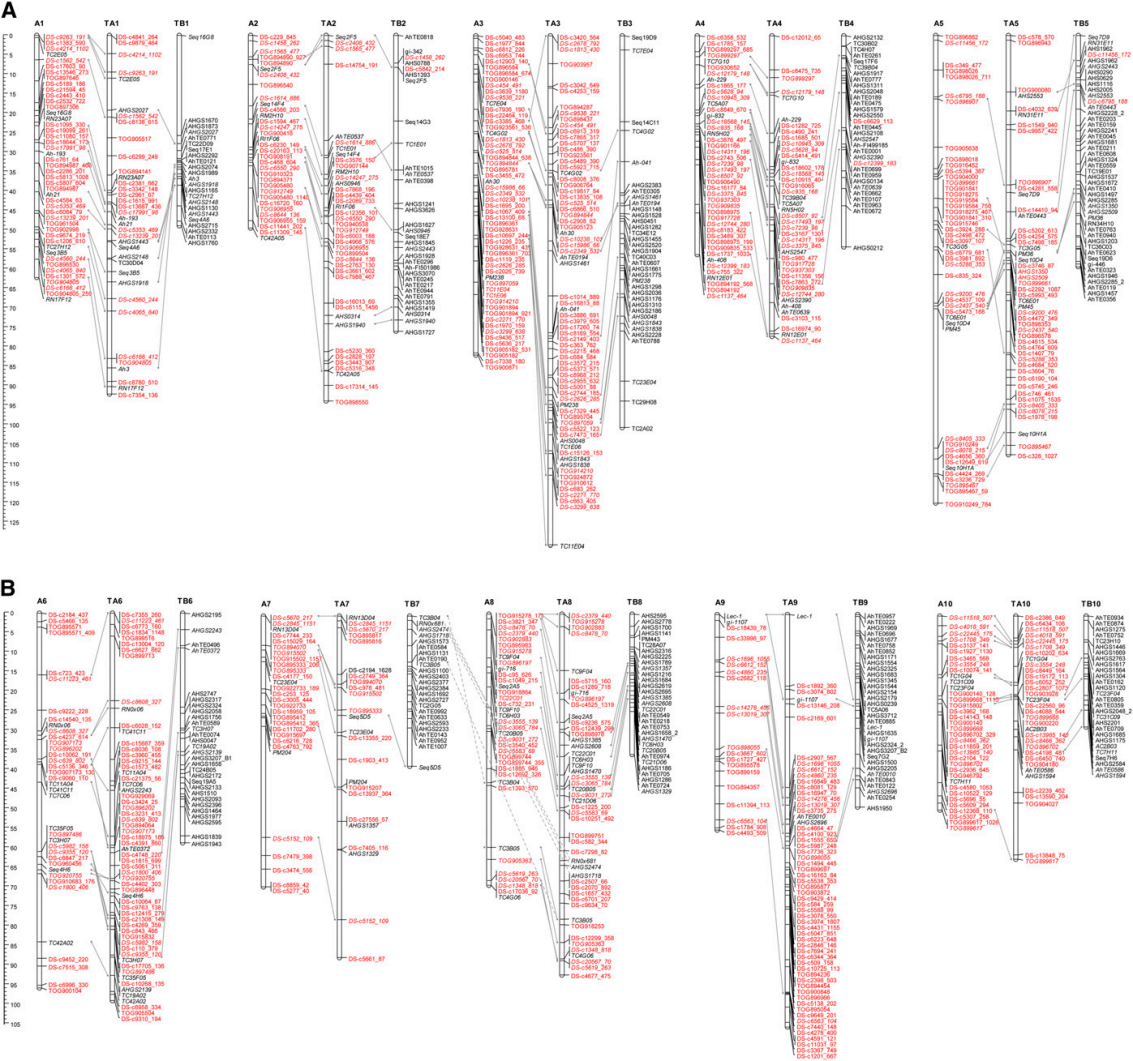
(A) Linkage maps of the diploid A and tetraploid AB *Arachis* genomes (linkage groups 1–5). The representation of the diploid A (linkage groups assigned AN) was generated using recombinant inbred lines derived from an *A. duranensis* × *A. stenosperma* cross. The representation of the tetraploid AB genome (linkage groups assigned TAN and TBN) was derived from a cross between cultivated peanut and an artificially induced tetraploid derived from two diploid wild species, *A. ipaënsis* and *A. duranensis*. Distances are centimorgans. Lines between linkage groups indicate marker correspondences and show genome syntenies. Single nucleotide polymorphism markers (SNPs) are represented in red; other markers, the incorporation of which were chosen to assign linkage groups and show synteny between A and B genomes, are shown in black. Note that almost all SNP markers are incorporated into the A genome of the tetraploid. (B) Linkage maps of the diploid A and tetraploid AB *Arachis* genomes (linkage groups 6–10). The representation of the diploid A (linkage groups assigned AN) was generated using recombinant inbred lines derived from an *A. duranensis* × *A. stenosperma* cross. The representation of the tetraploid AB genome (linkage groups assigned TAN and TBN) was derived from a cross between cultivated peanut and an artificially induced tetraploid derived from two diploid wild species, *A. ipaënsis* and *A. duranensis*. Distances are in centimorgans. Lines between linkage groups indicate marker correspondences and show genome syntenies. SNPs are represented in red; other markers, the incorporation of which was chosen to assign linkage groups and show synteny between A and B genomes, are shown in black. Note that almost all SNP markers are incorporated into the A genome of the tetraploid.

Using a minimum LOD score of 3.0 and a maximum recombination fraction of 0.35, 384 markers mapped into 10 linkage groups. These markers included 326 SNPs and 58 microsatellites. The map covered a total distance of 705.1 cM (Figure 4, A and B). Groups ranged from 49.5 cM to 120.1 cM, with an average distance of 1.84 cM between adjacent markers. Linkage groups were numbered according to the LG numbers of the A genome map (Moretzsohn *et al.* 2005; Leal-Bertioli *et al.* 2009) by the identification of syntenic markers.

#### Map for the tetraploid genome:

Using a minimum LOD score of 3.0 and a maximum recombination fraction of 0.35, 772 markers mapped into 20 linkage groups. Of these, 460 markers mapped into 10 linkage groups corresponding to the A genome component, and 312 markers mapped into the 10 B genome linkage groups. The 460 A genome markers included 360 SNPs and 100 microsatellites, whereas the B markers included 6 SNPs and 306 microsatellites. The map covered a total distance of 1487.3 cM, with 941.9 cM and 545.4 cM for the A and B genome components, respectively (Figure 4, A and B). Groups ranged from 62.2 cM to 127.1 cM, with an average distance of 2.05 cM, on the A genome and from 28.4 cM to 97.8 cM, with an average of 1.75 cM between adjacent markers, on the B genome component. Linkage groups were numbered according to syntenic markers as compared to the A diploid map.

#### Length of linkage maps:

The total genetic distances of our maps that were constructed using Mapmaker and the Kosambi mapping function were longer than those produced by Shirasawa *et al.* (2013), which were constructed using JoinMap with the Haldane mapping function. Generally, the Haldane mapping function provides longer distances than the Kosambi function, so the use of different mapping functions could not account for the differences. Initially, we had calculated the genetic distances in Mapmaker without genotyping error correction. The discrepancies between the total distances in these initial versions of our maps and the maps of Shirasawa were very considerable: 2445.4 *vs.* 1442 cM for the tetraploid and 987.6 *vs.* 544 cM for the diploid. When Mapmaker was run with error correction, the discrepancies were reduced and became small for the tetraploid map (1487.3 *vs.* 1442 cM) but remained substantial for the diploid A map (705.1 *vs.* 544 cM).

We investigated whether the remaining differences were attributable to different numbers of recombination events implicit in the different maps and/or were attributable to the influence of the different algorithms used in the mapping programs. First, using Excel sheets, we calculated estimations of the numbers of recombination events implicit in the two maps. Exact comparisons were difficult because of the large number of dominant markers in the Shirasawa map, and because of different error rates for the different types of markers used. However, the numbers of recombination events for the different maps seem similar. Then, we investigated the influence of the different mapping algorithms. We recalculated linkage group distances using Mapmaker and both Kosambi and Haldane functions, but using the marker orders obtained by Shirasawa *et al.* (2013). The newly calculated distances were longer than the ones obtained using JoinMap. To discard the possibility that the differences were attributable to the presence of both co-dominant and dominant markers in the Shirasawa map, we reanalyzed their data using only the co-dominant markers and, again, differences were obtained. We also reordered the markers with Mapmaker. This usually provided a result somewhat different from the JoinMap orders. The calculated differences decreased with these reordered markers, but they were still longer than distances calculated using JoinMap (results not shown).

#### Synteny analysis:

A total of 155 common markers were mapped in both the A and the tetraploid genome maps, while 59 were common to the A and B genome components of the tetraploid map (Figure 4, A and B). The 10 A genome linkage groups showed direct correspondences in both maps and the great majority of the loci were mapped in the same order. Markers were also generally collinear in both genome components of the tetraploid map, but linkage group B7 showed common markers to both A7 and A8 linkage groups. Direct correspondences were observed for all the other linkage groups. The linkage maps and genotyping data are available in an Excel sheet (File S1).

## Discussion

SNP markers are attractive for use in genetic mapping and marker-assisted breeding because they can be scored in parallel assays at favorable costs. Genotyping assays that detect SNPs are highly suitable to diploid plants. The assays, which are designed to detect a single locus (a DNA base) within the haploid genome, can efficiently distinguish the different combinations of bases present in the diploid genome (*e.g.*, GG, GT, TT). The application of SNPs in tetraploid plants is more difficult because the assays often detect four DNA bases (Akhunov *et al.* 2009).

Peanut is a crop with an allotetraploid genome of the type AABB (Husted 1936; Smartt *et al.* 1990; Fernández and Krapovickas 1994). It is estimated that the two genomes diverged approximately 3–3.5 million years ago (Nielen *et al.* 2012; Moretzsohn *et al.* 2013). Examination of A–B homeologous genome regions at the DNA sequence level reveals conserved segments of high sequence identity (approximately 95%), punctuated by *indel* regions without significant similarity, often consisting of repetitive DNA (Bertioli *et al.* 2009; 2013). Sequence identity within genes is even higher (approximately 97%). This study confirmed that, as indicated in previous studies (Burow *et al.* 2001; Moretzsohn *et al.* 2009; Foncéka *et al.* 2009; Guo *et al.* 2012; Shirasawa *et al.* 2013), A and B genomes are highly syntenic and most linkage groups present single correspondences and collinear marker order. For the markers that are in common with those of Shirasawa *et al.* (2013), the orders generated here are very similar; however, overall, colinearity between the A and B genomes is somewhat higher. This most likely indicates a slight improvement in accuracy of marker order because of the iterative use of the “ripple” command in the Mapmaker software. The maps generated here are longer than those obtained by Shirasawa *et al.* (2013), and this difference is small for the tetraploid map (1487.3 *vs.* 1442 cM) but is substantial for the diploid A map (705.1 *vs.* 544 cM). These differences cannot be explained by an increase in the number of predicted recombination events but are apparently attributable to the different software used, with Mapmaker used in this study and JoinMap used by Shirasawa *et al.* (2013). Several studies have shown that maps constructed with JoinMap are shorter than those constructed with Mapmaker (Sewell *et al.* 1999; Butcher *et al.* 2002; Tani *et al.* 2003; Gawłowska *et al.* 2005; Van’t Hof *et al.* 2008). Although the source of these differences is not completely clear, different algorithms for the treatment of genotyping errors seem to be the most likely cause. The large-scale exception in colinearity between the A and B maps observed here was also observed previously (Foncéka *et al.* 2009; Moretzsohn *et al.* 2009; Guo *et al.* 2012; Shirasawa *et al.* 2013). It indicates an apparent translocation event between LGs B7 and B8. Because this rearrangement was initially observed in a comparison between linkage maps developed for diploid species with A and B genomes (Moretzsohn *et al.* 2009), this chromosomal rearrangement must have happened before the tetraploidization of the cultivated peanut.

Here, we used a previously developed 1536 Illumina assay (Nagy *et al.* 2012) in diploid and tetraploid RIL mapping populations. The diploid population is derived from a cross of two wild A genome species, *A. duranensis* and *A. stenosperma*. The tetraploid population is derived from the cultivated peanut crossed with an artificially induced tetraploid [*A. ipaënsis* × *A. duranensis*]^4×^. These two populations provide an excellent genetic environment for study because the diploid is genetically simple, and the structure of the tetraploid population allows the A and B homeologs to be assigned. Although the SNPs were discovered using *A. duranensis* accessions not used for making these mapping populations (PI 475887 and Grif 15036), many of the markers did transfer to both populations used in this study. Transfer of markers was significantly higher for the tetraploid population (394) than the diploid population (329). This shows that the *A. duranensis vs. A. duranensis* comparison used for SNP discovery is more similar to the *A. duranensis* V14167 *vs. A. hypogaea* A genome comparison in the tetraploid population than to *A. duranensis* K7988 *vs. A. stenosperma* V10309 comparison in the diploid population used here.

Signal intensity plots from our A genome diploid mapping population had high average genotyping quality (GenTrain) scores, and the signal intensity plots were generally easy to interpret, with good separation between genotype calls (Figure 1A). The frequency distribution in GenTrain scores in the diploid shows a single high-scoring peak (at 0.7–0.79) (Figure 2). In the tetraploid, as expected, most of the polymorphic markers provided signal intensity plots that were distorted compared to diploid patterns and that were incorrectly scored using default parameters (Figure 3A). However, these scorings were easily manually corrected using the GenomeStudio software (Figure 3B). The degree of distortion in the tetraploid plots was variable, and the markers showed two distinct peaks in the frequency distribution of quality scores (Figure 2).

To explain this distribution of GenTrain scores in the tetraploid genotyping, we can envisage a scenario when the common ancestral species of the A and B genomes both harbored a monomorphic base at one locus in their genomes, *e.g.*, G. During evolution, a mutation may occur at this locus. This may happen in either the A or the B genome lineages, but it is very unlikely to happen in both. For this scenario, let us consider a G-to-T substitution in the A genome. Considering this polymorphism is present in our tetraploid population, this locus on the A genome will then have two possible allelic states, G or T. However, the homeologous position on the B genomes will harbor the monomorphic ancestral state with only one possible allelic state, G. In the mapping population, the genotypic combinations possible will be GG:GG, TG:GG, and TT:GG. In RIL populations, as in the case here, the heterozygous genotype is rare, making the genotype assays easier to score. Most of the signal intensity plots we observed were distorted in a way that was consistent with this type of scenario. The excess of one base (in this scenario, G) shifts one of the genotype clusters and reduces the separation between the groupings (Figure 1B). However, the degree of distortion was variable and formed two peaks in a frequency distribution of Gentrain scores (Figure 2). Genotyping assays with quality scores near the lower-scoring peak in frequency distribution (0.2–0.29) possibly detect bases on the A and B genomes with equal efficiency. Genotyping assays with quality scores near the higher-scoring peak in frequency distribution (0.5–0.59) possibly detect bases on one subgenome (here, almost always the A genome) with significantly greater efficiency than the other (presumably because of other A or B genome-specific substitutions or indels at or near the locus being assayed). In either case, the manual correction of tetraploid genotyping was simple; the software allows the calls to be adjusted for each marker on the whole population in a few steps (Figure 3). As for mapping populations with a greater proportion of heterozygous individuals (such as F_2_ populations), we anticipate that approximately 169 (40%) of the polymorphic assays had sufficiently low distortion that they would provide high-quality scores of all possible genotypes. SNP genotyping in the autotetraploid potato, in which five genotypes from nulliplex to quadruplex are possible, recently has been implemented with new algorithms (Voorrips *et al.* 2011) and features in GenomeStudio (Tech Note 970-2011-001). However, only three genotypes would be expected in allotetraploid peanut RIL populations with some residual heterozygosity because chromosome pairing is almost exclusively bivalent and only one of the two subgenomes would contain alternate alleles.

Genotyped markers in both the diploid and tetraploid populations had very high success in mapping into the linkage maps (Figure 4). Generally, the markers mapped with small genetic distances to their flanking markers. This shows that genotyping errors were low, because errors artificially increase distances between markers. In the diploid population, markers were distributed on all linkage groups. In the tetraploid, markers were almost exclusively placed on the A genome linkage groups and scattered throughout the 10 linkage groups (Figure 4). If only these SNP markers had been used for linkage map construction, then an A genome diploid map would have been constructed within a tetraploid context! This indicates that the scenario described in the previous paragraph is a good model for the origin of most polymorphisms. SNPs are highly diagnostic of either the A or the B genome clades. It also provides very graphic evidence that further supports the highly diploid genetic behavior of allotetraploid peanut.

The subgenome specificity of SNPs points to another possible utility, aiding the assembly of the cultivated peanut genome, which is currently being sequenced (http://www.peanutbioscience.com/peanutgenomeinitiative.html). One of the difficulties in this project arises from the similarity of the A and B genomes of the cultivated peanut. This similarity causes problems during assembly in which reads from the A and B genomes are falsely assembled into the same contig, causing errors and breaks in the genome assembly. One possible solution is the use of sequencing technologies that produce longer sequence reads. Another, perhaps complementary, solution would be to use knowledge of A and B genome-specific SNPs to segregate reads into A types or B types before assembly. The extreme genome specificity of the SNPs assayed here supports the feasibility of this approach.

Cultivated peanut has a very narrow genetic base and this has hampered the progress of genetic studies using cultivated × cultivated crosses. Our results here show that genotyping and map construction using SNPs in cultivated peanut are likely to be feasible. However, the discovery of a sufficient number of SNPs present in cultivated germplasm will be very challenging. The SNP assay used here would be of very limited use for cultivated peanut: only 88 SNPs were polymorphic across a panel of 70 cultivated *A. hypogaea* inbreds (P. Ozias-Akins, unpublished results). Furthermore, of these 88, only 42 showed a minor allele frequency more than 0.04. The narrow genetic base of cultivated peanut also has limited the gains obtained for some agronomic traits of interest in breeding programs that use only cultivated germplasm. This has stimulated interest in wild species that are a rich source of new alleles and new traits. However, wild species are not agronomically adapted and most wild alleles need to be eliminated by backcrossing to obtain a peanut cultivar. In this context, foreground and background genotyping schemes are particularly attractive in progeny derived from cultivated × wild crosses. Selected SNPs from the assay used here can be effectively used to monitor wild introgressions into cultivated peanut on the A genome. To generate an assay for monitoring wild introgressions on the B genome, SNPs identified in a panel of wild B genome species could be used to construct an informative assay. Together with the A genome SNPs identified here, this would allow the whole genome monitoring of wild allele introgression into cultivated peanut on a scale, and at a cost, that was previously impossible.

## Supplementary Material

Supporting Information
