# Supplementary material for: The Use of SNP Markers for Linkage Mapping in Diploid and Tetraploid Peanuts
Source: G3 (Bethesda). 2013 Nov 8;4(1):89–96. doi: 10.1534/g3.113.007617 (PMC3887543; doi:10.1534/g3.113.007617)
Supplement: Supporting Information [file supp_4_1_89__index.html]

The Use of SNP Markers for Linkage Mapping in Diploid and Tetraploid Peanuts — Supporting Information 

# The Use of SNP Markers for Linkage Mapping in Diploid and Tetraploid Peanuts

## Supporting Information for Bertioli *et al.*, 2014

**Files in this Data Supplement:**

- File S1 - Diploid and tetraploid maps (.xlsx, 495 KB)
